# Time-related changes in bacterial air contamination of sterile covered items in operating rooms: a systematic review and meta-analysis

**DOI:** 10.1186/s13756-026-01764-1

**Published:** 2026-05-13

**Authors:** Camilla Wistrand, Elisabeth Westerdahl, Ann-Sofie Sundqvist

**Affiliations:** 1 University Health Care Research Center, Faculty of Medicine and Health, Örebro University, Örebro, Sweden; 2 Department of Cardiothoracic and Vascular Surgery, Örebro University Hospital, O-house, 4th floor, Örebro, 701 85 SE Sweden; 3 Faculty of Medicine and Health, School of Health Sciences, Örebro University, Örebro, Sweden

**Keywords:** Bacterial air contamination, Colony forming units, Infection control, Meta-analysis, Operating room, Sterile covers, Surgery, Surgical site infection, Systematic review

## Abstract

**Background:**

There is still only limited evidence regarding how bacterial air contamination of sterile items changes over time in the operating room, even when protected by sterile covers. This review aimed to synthesize the available data to inform safe handling and preparation of sterile equipment.

**Methods:**

A systematic search was conducted for controlled studies that assessed time-related bacterial contamination of covered sterile items in operating rooms and that reported the outcome as colony forming units (CFU) measured at two or more time points, generating distinct timelines. Six databases were searched from inception to 1 September 2025: Ovid MEDLINE, the Cochrane Central Register of Controlled Trials, CINAHL, the Cochrane Database of Systematic Reviews, Embase, and Web of Science. A meta-analysis was performed using linear regression with CFU as the dependent variable and time (minutes) as the independent variable. The review followed PRISMA guidelines and was registered in PROSPERO (CRD420251018280).

**Results:**

Five timelines from four studies evaluating CFU counts yielded an R^2^ of 0.085, indicating that time explained 8.5% of CFU variation. Similarly, analysis of eight timelines from four studies assessing mean CFU values produced an R^2^ of 0.070, attributing 7% of variation to time. Neither analysis demonstrated a statistically significant linear association between time and CFU count (*p* = 0.176, CI: -1.319 < β < 4.863) or mean CFU values (*p* = 0.108, CI: 0.191 < β < 0.420) when sterile covers were used. Three additional timelines excluded from the meta-analysis showed mixed results: two indicated a positive association, while one showed no relationship.

**Conclusions:**

Time has only a modest impact on bacterial contamination of sterile items when protective covers are used, with minimal increases in CFU over different waiting periods. Clinically, this confirms that properly covered sterile items can be prepared in advance without compromising microbial safety, thus supporting efficient operating room organization and workflow. These findings reinforce the effectiveness of sterile covers as a key measure to reduce the risk of surgical site infections and ensure the safe handling of sterile equipment. Although covered storage appears safe, minimizing unnecessary waiting time, even with coverage, is good clinical practice.

**Supplementary Information:**

The online version contains supplementary material available at 10.1186/s13756-026-01764-1.

## Background

Infection prevention and control is a practical, evidence-based approach aimed at safeguarding both patients and healthcare workers from avoidable and preventable infections [1]. Surgical site infections (SSIs) are among the most frequent and serious complications following surgical procedures, leading to increased patient morbidity and imposing a substantial burden on healthcare systems [2]. A range of strategies can be employed to prevent SSIs, including strict adherence to evidence-based hygiene protocols, comprehensive training and education of healthcare personnel [3], and the implementation of systematic infection surveillance programs [4]. Specific preventive measures include maintaining optimal patient body temperature, blood glucose control, rigorous surgical hand disinfection, the use of sterile materials, and controlled operating room (OR) ventilation [5, 6].

The quality of air within the OR has long been recognized as a critical factor in SSI prevention, as airborne bacteria can contribute to infections during surgical procedures [7, 8], and evidence suggests that reducing airborne bacterial load is associated with a decrease in SSI rates [8]. In Sweden, ORs are equipped with specialized ventilation systems that are independent from the rest of the hospital infrastructure. These systems are designed to maintain air cleanliness at a level of ≤ 10 colony forming units (CFU) per m^3^, with a recommended target mean of 5 CFU/m^3^ [9]. To achieve this, the ventilation system employs high-efficiency particulate air filtration to reduce bacterial load, regulates air distribution by ensuring a minimum of 16 air changes per hour, and maintains positive air pressure within the OR to prevent the influx of less-clean air from adjacent areas [9, 10].

Air quality in the OR is influenced by several factors. The airborne bacteria predominantly originate from individuals present in the OR environment [11]. Increased personnel presence in the OR can lead to higher bacterial shedding into the air [12–14]. Smaller ORs may have lower air quality due to reduced air volume, which limits the dilution of airborne bacteria [15]. Additionally, frequent door openings can disrupt the positive air pressure, further compromising air cleanliness [16]. The movement of individuals within the OR also plays a critical role; calm and controlled movements are recommended to minimize disturbances of air quality [17, 18]. To uphold stringent hygiene standards, sterile surgical items must be prepared under controlled conditions and subsequently covered with sterile drapes to protect them from bacterial air contamination [17, 18].

We demonstrated in a recent systematic review that covering sterile items significantly reduced bacterial air contamination compared to leaving them uncovered, with consistent results observed across multiple time points ranging from 30 min to 24 h [19]. In the present review, we aimed to investigate whether bacterial contamination increases over time during the use of sterile covers. Understanding the potential escalation of contamination while sterile items are in use is critical, as even minor increases in bacterial load can compromise sterility, elevate the risk of SSI, and adversely affect patient outcomes. This knowledge is essential for optimizing the timing and protocols for OR preparation, ultimately enhancing patient safety and surgical quality.

Currently, there is a lack of consolidated evidence regarding the duration that sterile surgical items can remain under protective covers before the sterility is compromised. To address this gap, the present study aimed to analyse time-related changes in bacterial air contamination of sterile items stored under sterile covers in ORs.

## Methods

This systematic review was guided by the Preferred Reporting Items for Systematic Reviews (PRISMA) (see Table SI in the supplementary file), and was registered with PROSPERO (CRD420251018280) [20].

### Search strategy

In collaboration with two specialized subject librarians, a comprehensive search was conducted across six databases: Ovid MEDLINE, the Cochrane Central Register of Controlled Trials (CENTRAL), CINAHL, the Cochrane Database of Systematic Reviews, Embase, and Web of Science. The search spanned from database inception to 1 September 2025, and utilized keywords related to contamination, surgical instruments, and time. Search terms were adapted to align with the indexing systems and syntax of each database. Details of the search strategies used in Ovid MEDLINE are provided in the supplementary file (Table SII).

Studies were included if they met the following criteria:

- Both randomized controlled trials (RCTs) and non-randomized controlled trials were eligible.
- Sterile items were defined as sets of items or fields prepared within the OR.
- The study reported the primary outcome of airborne bacterial contamination in any form of CFU.
- Sterile items were covered with sterile drapes between a minimum of two distinct time points, to create a timeline.
- Various bacterial sampling methods were permitted, but data collection was restricted to the OR environment.
- No time limits were imposed on the duration of coverage.

## Data selection

**Fig. 1:**
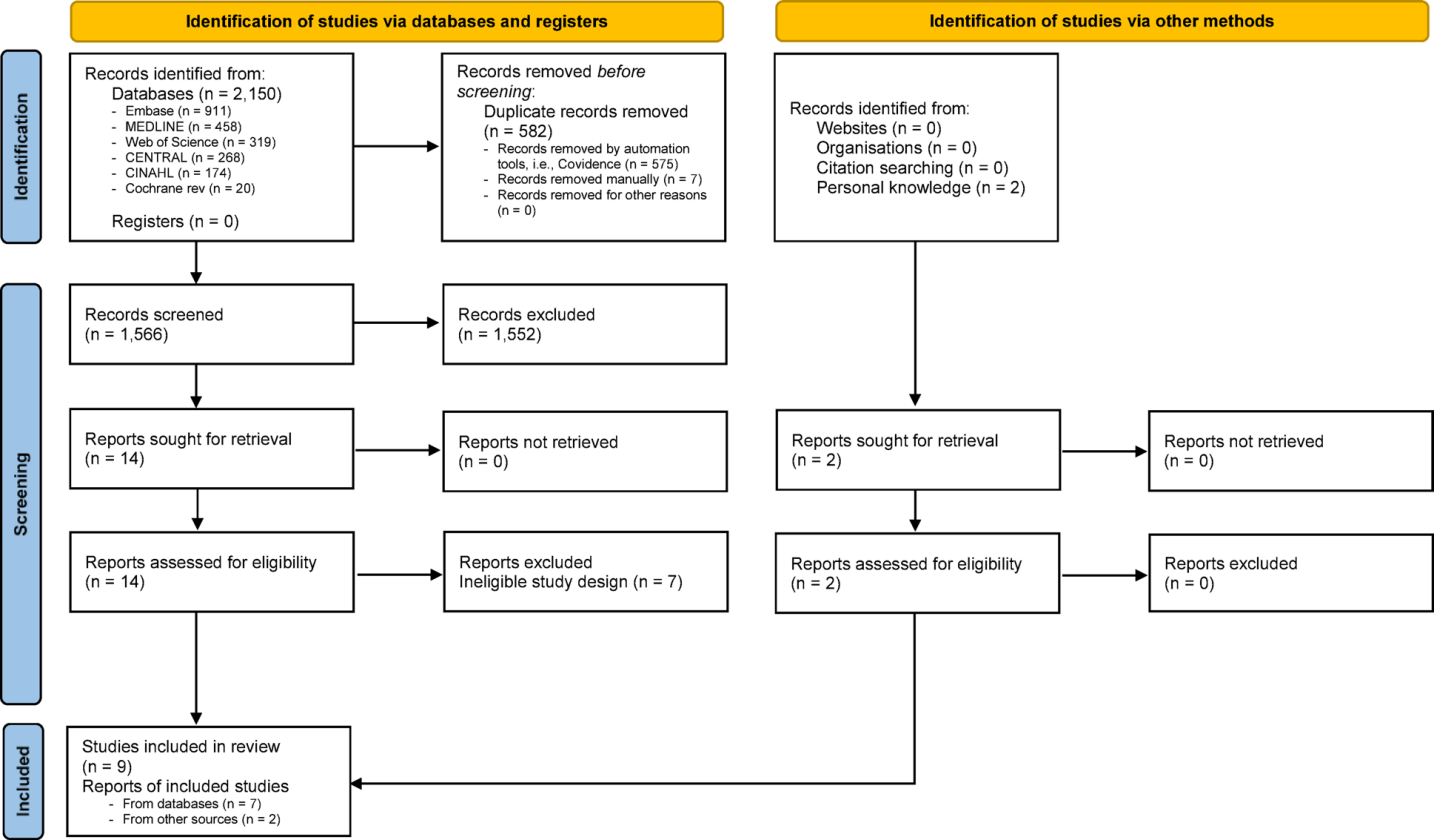
PRISMA 2020 flow diagram of the study selection process

Initial removal of duplicate records was performed by specialized subject librarians using EndNote X20.0.1. Subsequently, the remaining records were imported into Covidence systematic review software [21] for further processing. Study eligibility was independently assessed by two authors (CW and ASU) using Covidence. The screening process involved two stages: (1) evaluation of titles and abstracts, followed by (2) full-text review of potentially relevant manuscripts. Additionally, reference lists of retrieved articles were manually reviewed to identify further relevant studies. Discrepancies between the two reviewers regarding study inclusion were resolved through discussion. In cases where consensus could not be reached, the third author (EW) was consulted to make the final decision. Studies were excluded if they lacked primary data or a clearly defined study design, such as books, book chapters, conference abstracts, comments, editorials, letters, and review articles. No geographical restrictions were applied during the selection process; however, the language of publication was limited to Danish, English, Norwegian, and Swedish. A detailed flowchart of the study selection process is provided in Fig. 1. Records excluded after full text reading due to ineligible study design are listed in Table SIII in the supplementary file.

## Assessment of methodological quality and risk of bias

The methodological quality and risk of bias of the included studies were independently evaluated by two authors (CW and ASU) using standardized critical appraisal tools developed by the Joanna Briggs Institute [22, 23]. All studies were included in the analysis regardless of their quality. Risk of bias was assessed using an appraisal tool and classified as low (> 70% of criteria met), moderate (50–70% of criteria met), or high (< 50% of criteria met). The risk of bias assessments are presented in the supplementary file (Table SIV for RCTs and Table SV for quasi-experimental studies). In addition to quality assessment, relevant data on study characteristics, including country, year of publication, study settings, and other key variables, were systematically extracted.

### Data analysis

Quantitative data extraction was performed with a focus on CFU counts, including percentages, mean and median CFU values, and all reported time points. The dependent variable was defined as CFU, while the independent variable was time (measured in minutes). Data extraction was conducted collaboratively by all contributing authors. The primary objective of this analysis was to investigate time-related trends in bacterial air contamination when using sterile covers as a protective intervention in the OR. Statistical analysis was performed using version 29 of SPSS (SPSS Statistics, IBM, Armonk, NY, USA). Two meta-analyses were conducted. In studies where the data required for meta-analysis were incomplete, the corresponding authors were contacted to obtain the missing information. Multiple timelines were extracted from the studies and analysed for time-dependent associations with CFU levels. Descriptive statistics were reported using absolute count, mean, standard deviation (SD) or median, and interquartile range (IQR). For the meta-analytical component, linear regression and correlation analysis were employed to assess relationships and trends across the datasets. Prior to analysis, the distribution of CFU values within each experimental series was assessed using visual inspection of histograms. Given the low absolute CFU counts and the presence of several zero values, both mean, SD and median with IQR were presented to provide a comprehensive description of central tendency and dispersion. Logarithmic transformation was considered; however, due to the low counts and limited range of values, transformation was not applied.

## Results

The initial literature search yielded 2150 records, in addition to two references identified from other sources based on expert knowledge. After removal of duplicates, 1570 titles and abstracts were assessed; 16 were considered suitable for full text review and assessment of eligibility regarding the inclusion criteria. Following the eligibility assessment, nine studies were included in this review: three from the USA [24–26], two from Iran [27, 28], two from Sweden [29, 30], and two from Turkey [31, 32]. The year of publication ranged from 1993 to 2023. All included studies reported a minimum of two measurement time points, with most incorporating multiple repeated measures (intervals). The shortest data collection interval was 0–120 min, which was observed in two studies [31, 32], while the longest spanned up to 7 days [26] (Table 1).

**Table 1:** Descriptive characteristics of the studies, including factors that might have influenced the results

Author (year) country of origin [reference number]	Design	Number of occasions	Time points of data collection	Type of ventilation system	Clothing material	Door openings	Number of individuals	Methods of sampling colony forming units	Bacteria isolated
Zarei et al. [27] Iran	Quasi-experimental intervention study without patients	27	After 15, 30, 45, 60, 120, 180, and 240 min, and 24 h.	Turbulent ventilation system. Air inflow of 600 L/sec, 15 air changes/hr.	Unknown	Unknown	2	Blood agar plates isolating bacterial air contamination (mean and SD).	Unknown
Zarei et al. [28] Iran	Quasi-experimental intervention study without patients	27	After 15, 30, 45, 60, 120, 180, and 240 min, and 24 h.	Turbulent ventilation system. Air inflow of 600 L/sec, 15 air changes/hr.	Unknown	Unknown	2	Blood agar plates isolating bacterial air contamination (mean and SD).	Unknown
Wistrand et al. [29] Sweden	Quasi-experimental intervention study without patients	24	After 4, 8, 12, 16, and 24 h.	Upward displacement ventilation system. Air inflow of 700 L/sec, 16 air changes/hr. Automatically decreased to 300 L/sec if no movements.	Individuals wore tightly woven reusable clothes, caps, facemasks, and the person preparing the sterile fields and was dressed in a sterile gown and sterile indicator gloves.	Door was opened at every time point for data collection, through air lock (*n* = 5).	Preparation of agar plates *n* = 2. Data collection *n* = 1.	Blood agar plates isolating bacterial air contamination (CFU per item).	*Cutibacterium acnes*, *Micrococcus luteus*, *Staphylococcus epidermidis*, *Staphylococcus warneri*, *Micrococcus flavus*, *Staphylococcus saprophyticus*, *Staphylococcus haemolyticus*
Uzun et al. [31] Turkey	Randomized controlled trial including patients	30	After 0, 15, 30, 60, 90, and 120 min.	Laminar ventilation system.	Unknown	No door openings allowed.	Unknown	K-wires were placed in a liquid culture medium. After 24-h incubation period, samples from liquid were cultured on blood agar using swabs (CFU/mL).	CoNS, *Staphylococcus aureus*, *Streptococcus agalactia*
Markel et al. [24] USA	Quasi-experimental intervention study without patients	9	After 4, 8, and 24 h.	High efficiency particulate air filtration system. 22 and 28 air changes/hr. OR size 126.5 m^2^ and 194.5 m^2^.	Study personnel wore standard hospital-issued clean scrub attire, head coverings, surgical masks, and shoe covers.	No door openings allowed.	Unknown	Blood agar plates (*n* = 162) isolating bacterial air contamination (median/m³ and IQR).	Unknown
Menekse et al. [32] Turkey	Randomized controlled trial including patients	22	After 0, 30, 60, 90, and 120 min.	Laminar ventilation system.	Unknown	Unknown	Unknown	Screws from implant sets (% of items contaminated).	Unknown
Sandström et al. [30] Sweden	Quasi-experimental intervention study without patients	30	After 12, 15, and 24 h.	Upward displacement ventilation system.	Study personnel wore cotton/polyester clothing and sterile gowns and gloves.	Door openings according to protocol.	2	Blood agar plates isolating bacterial air contamination (CFU per item).	CoNS, *Micrococcus*
Dalstrom et al. [25] USA	Randomized controlled trial without patients	15	Every 30 min for 4 h.	Positive air inflow.	Unknown	Unknown	Unknown	Swabs (% of items contaminated).	Unknown
Campbell et al. [26] USA	Quasi-experimental intervention study without patients	4 and 2	At start before coverage and every day for 4 days. Second timeline at start before coverage and after 7 days.	Unknown	Unknown	Unknown	Unknown	RODAC plates rubbed onto the surface. Cultures *n* = 100 and *n* = 80 (CFU per item).	CoNS, *S. aureus*, *Micrococcus*,* Corynebacterium sp.*

CFU = colony forming unit, CoNS = coagulase negative staphylococci, SD = standard deviation

The meta-analysis was conducted in two distinct components based on characteristics of the outcome data: one assessing CFU reported as absolute counts, and the other assessing CFU reported as mean ± SD. Two of the studies reported both metrics [29, 30], and hence were included in both analyses. Three studies were not included in the meta-analyses due to reporting outcomes as medians or percentages rather than CFU counts or means ± SD [24, 31, 32]; these were analysed separately and summarized narratively with statistical context where applicable. Two studies contributed multiple independent timelines [26, 28], resulting in a total of 11 timelines.

**Fig. 2:**
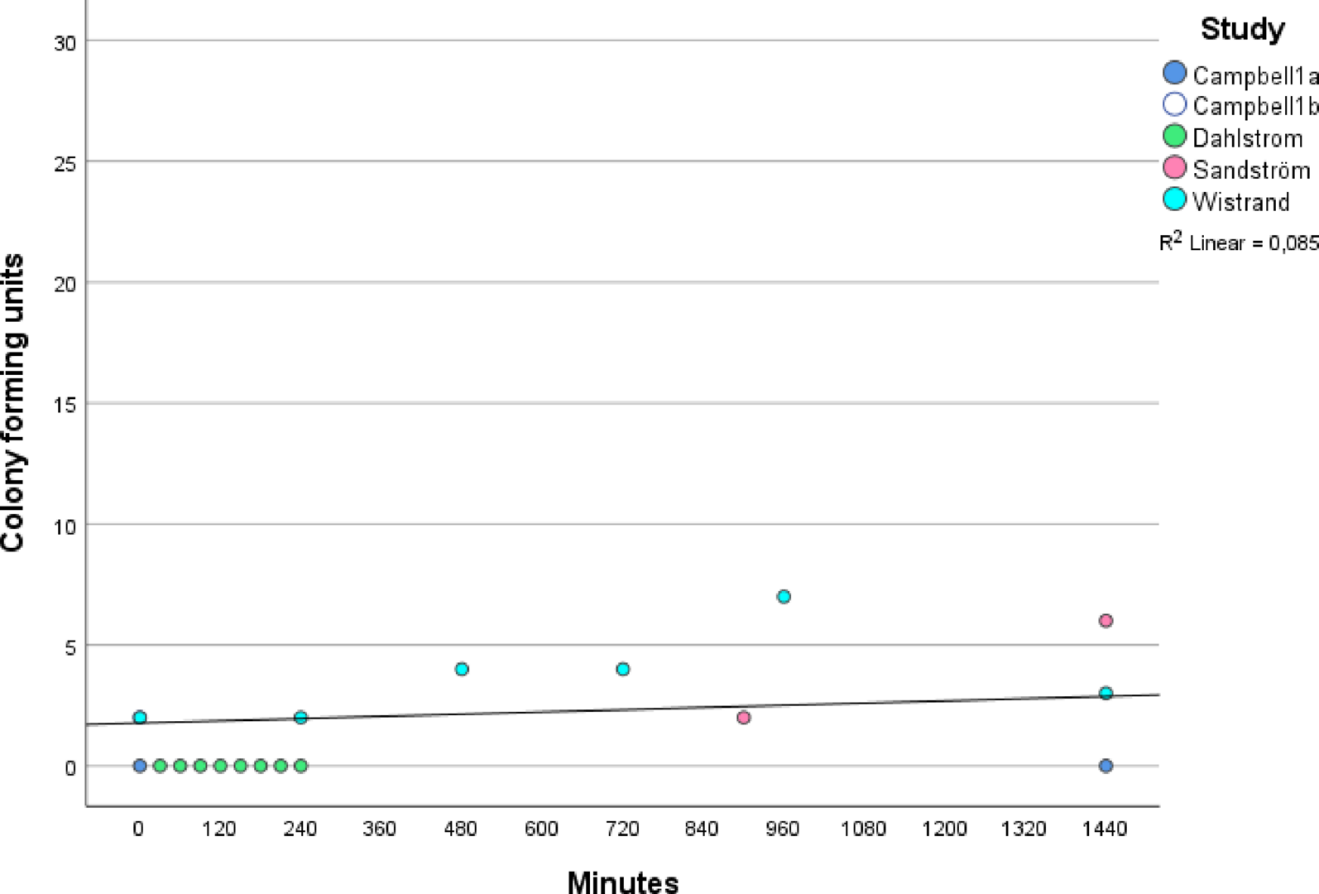
Meta-analysis of time and bacterial contamination in number=counts of colony forming units. Trend analysis was performed using linear regression within a meta-analytic framework. The coefficient of determination (R^2^) indicates the proportion of variance explained by the model, with values near 1 reflecting a strong relationship between colony forming units and time. Five timelines from four independent studies were included, with Campbell 1a and 1b representing separate timelines within the same study

In the meta-analysis of five timelines assessing CFU counts, the coefficient of determination (R^2^) was 0.085, indicating that 8.5% of the variation in CFU could be attributed to time (Fig. 2).

**Fig. 3:**
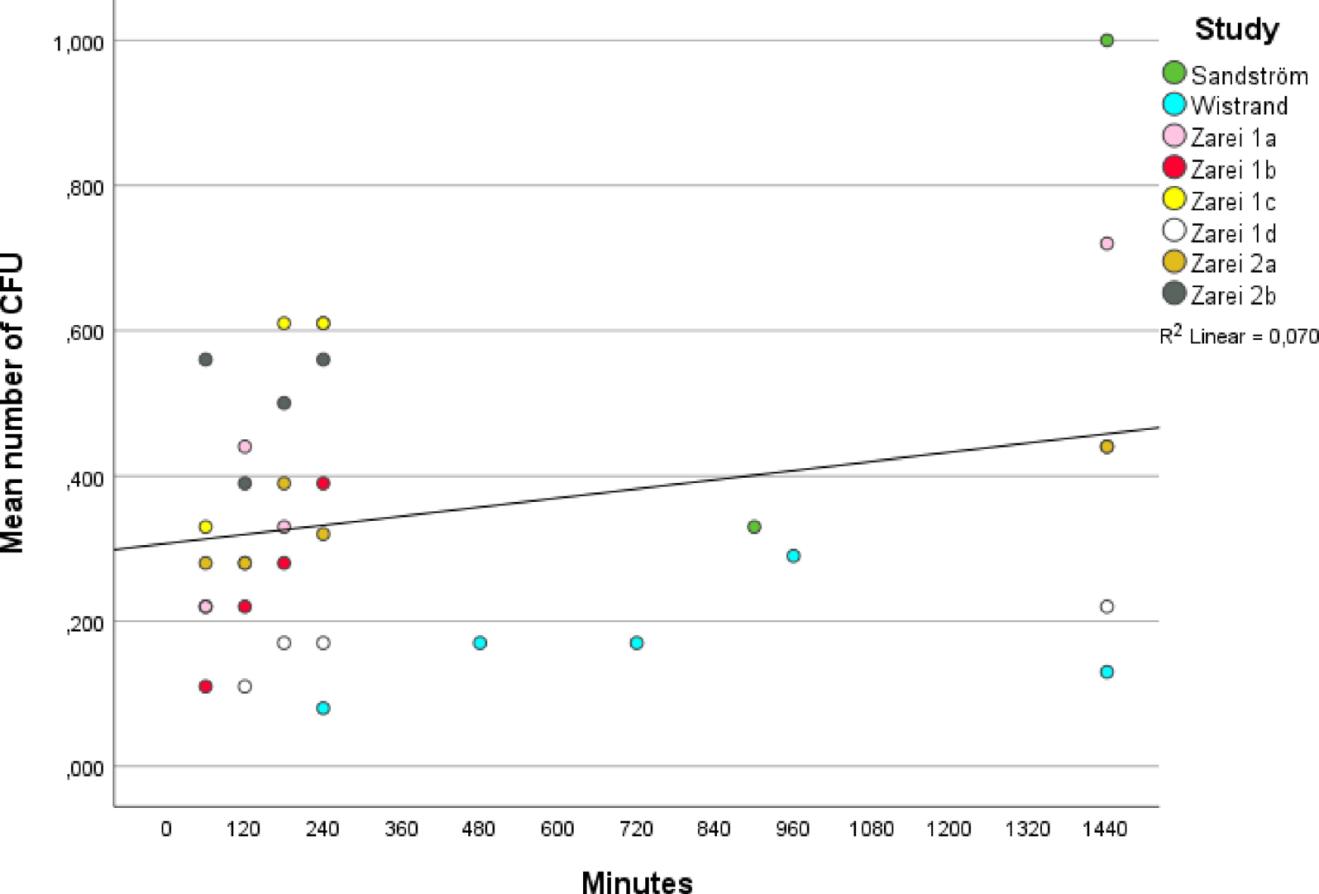
Meta-analysis of time and bacterial contamination in mean colony forming units. The coefficient of determination (R^2^) indicates the proportion of variance explained by the model, with values near 1 reflecting a strong relationship between colony-forming units (CFU) and time. The meta-analysis included eight timelines from four studies, with two studies by Zarei et al. contributing multiple timelines

In the analysis of eight timelines evaluating mean CFU values, R^2^ was 0.070, suggesting a 7% explanatory power for time (Fig. 3).

Neither analysis demonstrated a statistically significant association between time and CFU count (*p* = 0.176, CI: − 1.319 < β < 4.863) or mean CFU value (*p* = 0.108, CI: 0.191 < β < 0.420) when sterile items were protected by sterile covers.

Markel et al. reported no increase in CFU count over time when sterile items were protected by sterile covers [24]. Median CFU/m³ values were consistently zero across three time points: 4, 8, and 24 h, with IQR also at zero, indicating no detectable bacterial contamination appearing over time [24]. Menekse et al. presented outcomes as binary growth/no-growth observations across five time points. No contamination was observed at 0 and 30 min. However, contamination was detected at 60 min, 90 min, and 120 min, suggesting a gradual increase in contamination over time [32].

**Fig. 4:**
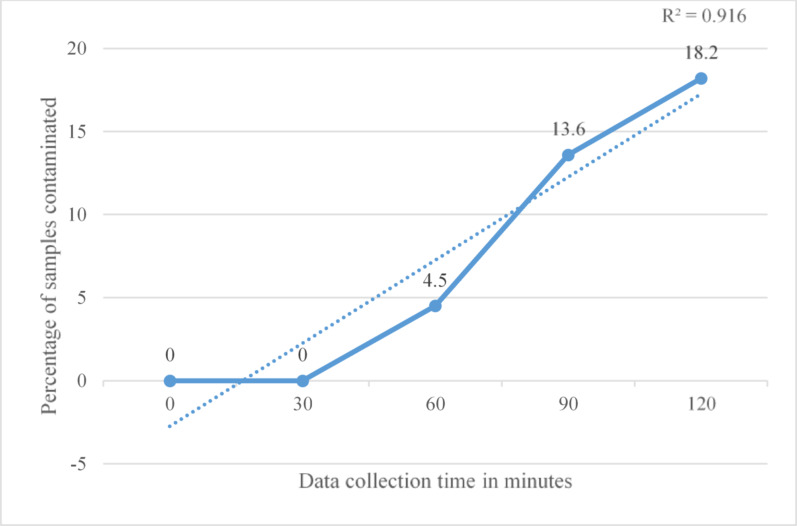
Association between time and bacterial contamination. The coefficient of determination (R^2^) indicates the proportion of variance explained by the regression model, with values near 1 reflecting a strong relationship between colony-forming units and time. This figure presents a single analysis based on the percentage of contaminated samples

**Fig. 5:**
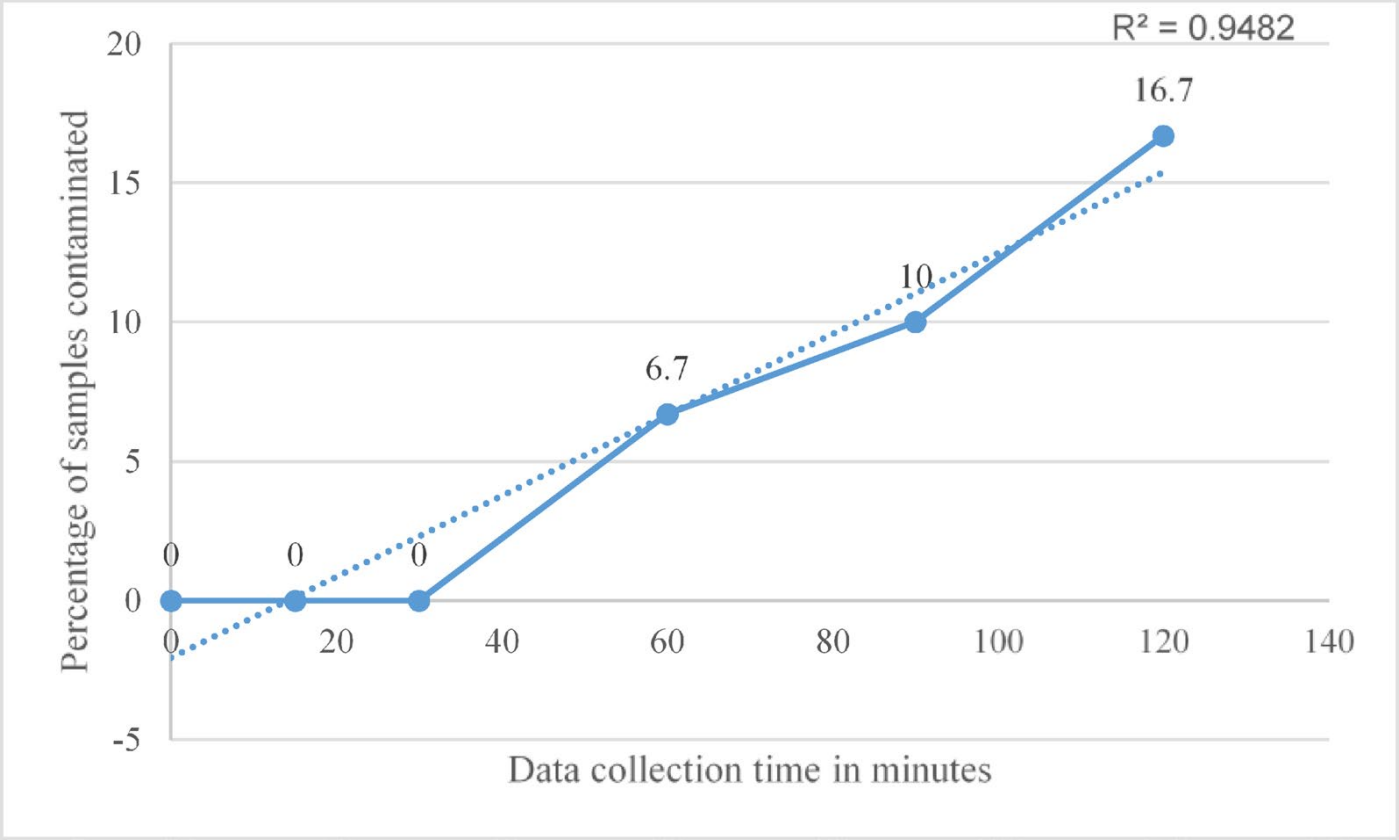
The association between time and bacterial contamination. The coefficient of determination (R^2^) indicates the proportion of variance explained by the regression model, with values near 1 reflecting a strong relationship between colony-forming units and time. This figure shows a single analysis based on the percentage of contaminated samples

Similarly, Uzun et al. reported no binary growth at 0, 15, and 30 min. Contamination was observed on items at 60, 90, and 120 min, indicating a comparable trend of increasing contamination with time [31] (Fig. 5).

## Discussion

The meta-analyses in this review demonstrate that time exerts only a modest effect on bacterial air contamination of sterile items protected by covers, explaining 8.5% of the variation in CFU counts (R^2^ = 0.085) and 7% of the variation in mean CFU values (R^2^ = 0.070). These findings suggest that, while contamination may increase slightly over time, the effect is limited when appropriate protective measures are in place; this highlights the continued importance of sterile covers in maintaining microbial safety during storage and handling. Historically, both national and international guidelines have lacked specificity regarding the permissible duration for which sterile surgical items may remain covered prior to use [17, 18]. Existing recommendations have generally advised that sterile items be prepared as close to the time of surgery as possible. In Sweden, this ambiguity has prompted more time-specific guidance. In response, the Swedish Association of Operating Room Nurses issued a national recommendation in 2025, advising that sterile surgical items may remain covered for a maximum of 15 h before use [33]. This recommendation was grounded in a body of evidence evaluating the effects of covering versus not covering sterile goods across various time intervals [19, 24, 26, 28–30, 32, 34–36]. However, caution is warranted when interpreting these guidelines, as environmental variability across OR settings may influence airborne bacterial load and contamination risk.

Of the 9 studies included, most (6 studies) had a low risk of bias, but the remaining studies had a moderate risk of bias. This indicates that the results should be interpreted with caution, as the methodological quality of the studies can affect the reliability of the conclusions. If all studies had been of high quality, it would have been easier to draw more robust conclusions. Weaknesses in the studies included the absence of baseline data regarding environmental factors that influence air quality. Specifically, some of the studies lacked information on the type and performance of ventilation systems [26], the clothing worn by personnel during data collection [25, 26, 28, 31, 32], the door-opening frequency [25, 26, 28, 32], and the number of individuals present during sampling [24–26, 31, 32]. These variables are known to affect airborne bacterial load, and may have influenced the reported outcomes. At the same time, the quality of the studies does not affect the design or methodology of our literature review, as its purpose was to compile and analyse the available evidence regardless of variations in study quality.

One limitation of our analysis was the inability to perform one unified meta-analysis across all included datasets, due to heterogeneity in outcome reporting. Our meta-analysis demonstrated a weak association between CFU counts and time when sterile fields or items were protected by sterile covers. However, these findings must be interpreted with caution due to the limited number and varied designs of the included studies. Although CFU was the common outcome measure, differences in how it was reported (e.g. counts, means, medians, or binary growth/no-growth formats) posed challenges for synthesizing the data into a unified meta-analysis. Consequently, three out of fourteen timelines were excluded from meta-analysis. Among the eleven timelines included in the meta-analysis, results consistently indicated a low association between bacterial contamination and time; this was in agreement with one of the three timelines excluded from the meta-analysis. In contrast, two of the studies excluded from the meta-analysis demonstrated a positive correlation between contamination and time. These conflicting findings highlight the lack of consensus in knowledge, and underscore the need for cautious clinical interpretation and the need for future research.

To enhance robustness, both meta-analyses incorporated two overlapping timelines that reported both CFU counts and mean CFU values [29, 30]. Despite efforts to obtain raw CFU count data from the corresponding authors of the studies excluded from meta-analysis, retrieval was unsuccessful due to various constraints.

The relatively small number of studies included in this review also limits the generalizability of the findings. Nonetheless, the results contribute valuable insights for healthcare professionals and support the ongoing discourse on optimizing sterile field management in surgical environments.

## Conclusions

Time has only a modest impact on bacterial contamination of sterile items when protective covers are used, with minimal increases in CFU counts over different waiting periods. Clinically, this confirms that properly covered sterile items can be prepared in advance without compromising microbial safety, thus supporting efficient OR organization and workflow. These findings reinforce the effectiveness of sterile covers as a key measure to reduce the risk of SSI and ensure the safe handling of sterile equipment. Although covered storage appears safe, minimizing unnecessary waiting time, even with coverage, is good clinical practice.

## Supplementary Information

Below is the link to the electronic supplementary material.

Supplementary Material 1

## Data Availability

The datasets used and analysed in this meta-analysis are available from the corresponding author on reasonable request. The study protocol and statistical analysis plan are accessible on PROSPERO under the registration number CRD42022323113 [20].

## Abbreviations

CFU: Colony forming unit
IQR: Interquartile range
OR: Operating room
RCT: Randomized controlled trial
SD: Standard deviation
SSI: Surgical site infection
