# Supplementary material for: Time-related changes in bacterial air contamination of sterile covered items in operating rooms: a systematic review and meta-analysis
Source: Antimicrob Resist Infect Control. 2026 May 13;15:70. doi: 10.1186/s13756-026-01764-1 (PMC13173913; doi:10.1186/s13756-026-01764-1)
Supplement: Supplementary file 1 — Supplementary Material 1 [file 13756_2026_1764_MOESM1_ESM.docx]

**Table SI.** PRISMA 2020 Checklist

| **Section and Topic** | **Item #** | **Checklist item** | **Location where item is reported** |  |  |
| --- | --- | --- | --- | --- | --- |
| **TITLE** | | |  |  |  |
| Title | 1 | Identify the report as a systematic review. | Line 2 |  |  |
| **ABSTRACT** | | |  |  |  |
| Abstract | 2 | See the PRISMA 2020 for Abstracts checklist. | Line 23 |  |  |
| **INTRODUCTION** | | |  |  |  |
| Rationale | 3 | Describe the rationale for the review in the context of existing knowledge. | Line 106 |  |  |
| Objectives | 4 | Provide an explicit statement of the objective(s) or question(s) the review addresses. | Line 108 |  |  |
| **METHODS** | | |  |  |  |
| Eligibility criteria | 5 | Specify the inclusion and exclusion criteria for the review and how studies were grouped for the syntheses. | Line 123 |  |  |
| Information sources | 6 | Specify all databases, registers, websites, organisations, reference lists and other sources searched or consulted to identify studies. Specify the date when each source was last searched or consulted. | Line 116  Figure 1  Line 118 |  |  |
| Search strategy | 7 | Present the full search strategies for all databases, registers and websites, including any filters and limits used. | Line 114 supplementary file (Table SII) |  |  |
| Selection process | 8 | Specify the methods used to decide whether a study met the inclusion criteria of the review, including how many reviewers screened each record and each report retrieved, whether they worked independently, and if applicable, details of automation tools used in the process. | Line 136 and forward. |  |  |
| Data collection process | 9 | Specify the methods used to collect data from reports, including how many reviewers collected data from each report, whether they worked independently, any processes for obtaining or confirming data from study investigators, and if applicable, details of automation tools used in the process. | Line 138 |  |  |
| Data items | 10a | List and define all outcomes for which data were sought. Specify whether all results that were compatible with each outcome domain in each study were sought (e.g. for all measures, time points, analyses), and if not, the methods used to decide which results to collect. | Line 123 |  |  |
|  | 10b | List and define all other variables for which data were sought (e.g. participant and intervention characteristics, funding sources). Describe any assumptions made about any missing or unclear information. | Table 1 |  |  |
| Study risk of bias assessment | 11 | Specify the methods used to assess risk of bias in the included studies, including details of the tool(s) used, how many reviewers assessed each study and whether they worked independently, and if applicable, details of automation tools used in the process. | Line 151  Line 153 |  |  |
| Effect measures | 12 | Specify for each outcome the effect measure(s) (e.g. risk ratio, mean difference) used in the synthesis or presentation of results. | Line 162 |  |  |
| Synthesis methods | 13a | Describe the processes used to decide which studies were eligible for each synthesis (e.g. tabulating the study intervention characteristics and comparing against the planned groups for each synthesis (item #5)). | Line 162 and forward |  |  |
|  | 13b | Describe any methods required to prepare the data for presentation or synthesis, such as handling of missing summary statistics, or data conversions. | Line 169 |  |  |
|  | 13c | Describe any methods used to tabulate or visually display results of individual studies and syntheses. | Line 172 and forward |  |  |
|  | 13d | Describe any methods used to synthesize results and provide a rationale for the choice(s). If meta-analysis was performed, describe the model(s), method(s) to identify the presence and extent of statistical heterogeneity, and software package(s) used. | Line 162  Line 174  Line 168 |  |  |
|  | 13e | Describe any methods used to explore possible causes of heterogeneity among study results (e.g. subgroup analysis, meta-regression). |  |  |  |
|  | 13f | Describe any sensitivity analyses conducted to assess robustness of the synthesized results. |  |  |  |
| Reporting bias assessment | 14 | Describe any methods used to assess risk of bias due to missing results in a synthesis (arising from reporting biases). | Line 152 |  |  |
| Certainty assessment | 15 | Describe any methods used to assess certainty (or confidence) in the body of evidence for an outcome. | Line 152 |  |  |
| **RESULTS** | | |  |  |  |
| Study selection | 16a | Describe the results of the search and selection process, from the number of records identified in the search to the number of studies included in the review, ideally using a flow diagram. | Figure 1 |  |  |
|  | 16b | Cite studies that might appear to meet the inclusion criteria, but which were excluded, and explain why they were excluded. | supplementary file  (Table SIII) |  |  |
| Study characteristics | 17 | Cite each included study and present its characteristics. | Table 1 |  |  |
| Risk of bias in studies | 18 | Present assessments of risk of bias for each included study. | supplementary file  (Table SIV) |  |  |
| Results of individual studies | 19 | For all outcomes, present, for each study: (a) summary statistics for each group (where appropriate) and (b) an effect estimate and its precision (e.g. confidence/credible interval), ideally using structured tables or plots. | Figure 2 to 5 |  |  |
| Results of syntheses | 20a | For each synthesis, briefly summarise the characteristics and risk of bias among contributing studies. | supplementary file  (Table SIV) (Table SV) |  |  |
|  | 20b | Present results of all statistical syntheses conducted. If meta-analysis was done, present for each the summary estimate and its precision (e.g. confidence/credible interval) and measures of statistical heterogeneity. If comparing groups, describe the direction of the effect. | Line 217 and forward  Figure 2 to 5 |  |  |
|  | 20c | Present results of all investigations of possible causes of heterogeneity among study results. |  |  |  |
|  | 20d | Present results of all sensitivity analyses conducted to assess the robustness of the synthesized results. | Line 286 |  |  |
| Reporting biases | 21 | Present assessments of risk of bias due to missing results (arising from reporting biases) for each synthesis assessed. | Table 1 |  |  |
| Certainty of evidence | 22 | Present assessments of certainty (or confidence) in the body of evidence for each outcome assessed. | Line 275 |  |  |
| **DISCUSSION** | | |  |  |  |
| Discussion | 23a | Provide a general interpretation of the results in the context of other evidence. | Line 294 |  |  |
|  | 23b | Discuss any limitations of the evidence included in the review. | Table 1  Line 259 |  |  |
|  | 23c | Discuss any limitations of the review processes used. | Line 272 |  |  |
|  | 23d | Discuss implications of the results for practice, policy, and future research. | Line 284 |  |  |
| **OTHER INFORMATION** | | |  |  |  |
| Registration and protocol | 24a | Provide registration information for the review, including register name and registration number, or state that the review was not registered. | Line 37  Line 316 |  |  |
|  | 24b | Indicate where the review protocol can be accessed, or state that a protocol was not prepared. | Line 112 |  |  |
|  | 24c | Describe and explain any amendments to information provided at registration or in the protocol. |  |  |  |
| Support | 25 | Describe sources of financial or non-financial support for the review, and the role of the funders or sponsors in the review. | Line 320 |  |  |
| Competing interests | 26 | Declare any competing interests of review authors. | Line 318 |  |  |
| Availability of data, code and other materials | 27 | Report which of the following are publicly available and where they can be found: template data collection forms; data extracted from included studies; data used for all analyses; analytic code; any other materials used in the review. | Line 314 |  |  |

*From:* Page MJ, McKenzie JE, Bossuyt PM, Boutron I, Hoffmann TC, Mulrow CD, et al. The PRISMA 2020 statement: an updated guideline for reporting systematic reviews. *BMJ* 2021; **372**: n71. doi: 10.1136/bmj.n71. For more information, visit: <http://www.prisma-statement.org/>

**Table SII.** Search strategy used in Ovid MEDLINE: ALL 1946 to 1 September 2025.

| **Concept** | **#** | **Search^1^** | **Results** |
| --- | --- | --- | --- |
| **Surgical instruments** | **1** | **exp Operating Rooms/** | **16,872** |
|  | **2** | **(operating room* or operation room* or Operation theater or operation theatre or Operating theatre or operating theater or preoperative preparation* or surgical preparation* or surgery preparation*).ab,kf,ti.** | **50,605** |
|  | **3** | **1 or 2** | **57,709** |
|  | **4** | **(sterile or setup or set-up or set up or setups or set-ups or set ups or Tray*).ab,kf,ti.** | **169,676** |
|  | **5** | **3 and 4** | **1,875** |
|  | **6** | **Surgical Instruments/** | **21,373** |
|  | **7** | **(instrument tray* or sterile tray* or surgical tray* or surgical instrument* or surgery instrument* or Sterile field* or Sterile good* or instrument table*).ab,kf,ti.** | **6,206** |
|  | **8** | **6 or 7** | **25,998** |
|  | **9** | **5 or 8** | **27,577** |
| **Time** | **10** | **exp Time Factors/** | **125,6008** |
|  | **11** | **(time* or earl*).ab,kf,ti.** | **7,000,553** |
|  | **12** | **10 or 11** | **7,769,960** |
| **Contamination** | **13** | **exp Equipment Contamination/ or exp Air Microbiology/ or Cross Infection/ or exp Surgical Wound Infection/ or Infection Control/ or exp Microbiology/ or exp Colony Count, Microbial/ or exp Bacteria/** | **1,799,764** |
|  | **14** | **(Equipment Contamination or Air Microbiology or Surgical Wound Infection* or Surgical site Infection* or Colony forming unit* or blood agar plate* or Culture or Swabs or ambient air or contamination or contaminating or Bacter* or Cross Infection* or Microbiology or CFU or postoperative infection* or postoperative wound infection* or post-operative infection* or post-operative wound infection* or infection control or surgical site infection* or surgical wound infection* or surgical infection*).ab,kf,ti.** | **2,112,242** |
|  | **15** | **13 or 14** | **3,087,771** |
| **Combined sets** | **16** | **9 and 12 and 15** | **507** |
| **Limits^2^** | **17** | **limit 16 to (danish or english or norwegian or swedish)** | **458** |
| ^1^ Date searched: Initial search 2023-11-08, rerun 2025-04-01, second rerun 2025-09-01 | | | |
| ^2^ Limits applied: Publications in English, Danish, Norwegian or Swedish language | | | |
| / = Mesh-term; * = truncation; exp = exploded Mesh-term; ab = abstract; kf = keyword heading word; ti = Title | | | |

**Table SIII.** Records excluded after full text reading due to ineligible study design

| 1 | Allen G. Evidence for practice. Time-dependent contamination of OR trays. *AORN J.* 2008;**88**(2): 288–9. |
| --- | --- |
| 2 | Bible JE, O’Neill KR, Crosby CG, Schoenecker JG, McGirt MJ, Devin CJ. Implant contamination during spine surgery. *Spine J.* 2013;**13**(6):637–40. |
| 3 | Chosky SA, Modha D, Taylor GJ. Optimisation of ultraclean air. The role of instrument preparation. *J Bone Joint Surg Br.* 1996;**78**(5):835–7. |
| 4 | Pirmoradian M, Pourbabaei A, Sangi MG, Kalhor N. A clinical trial of contamination of surgical instruments with Staphylococcus aureus during long time orthopedic surgeries. *Qom Uni Med Sci J.* 2013;**7**(3):26–9. |
| 5 | Qvistgaard M, Almerud-Österberg S, Lovebo J. Covering surgical instruments with single- or double-layer drape pending surgery: an experimental study in a perioperative setting. *J Infect Prev.* 2021;**22**(3):126–31. |
| 6 | Shisoka J, Mutisya A, Makworo D. Duration of time to microbial colonization on surgical instruments and surgical sites intraoperatively at a public referral hospital Kenya. *East Afr Med J*. 2023;**100**(9):6234–42. |
| 7 | Wistrand C, Söderquist B, Friberg Ö, Sundqvist AS. Bacterial air contamination and the protective effect of coverage for sterile surgical goods: a randomized controlled trial. *Am J Infect Control*. 2025;**53**(4):467–72. |

**Table SIV.** Overview of the risk of bias assessment for the randomized controlled trials included in the review.

| **Quality appraisal statements^1^** | | **Author (year) [reference number]** | | |
| --- | --- | --- | --- | --- |
|  |  | **Dalstrom et al. (2008) [25]** | **Uzun et al. (2019) [31]** | **Menekse et al. (2015) [32]** |
| 1. Was random assignment used for treatment groups? | | yes | yes | yes |
| 2. Was group allocation concealed? | | no | yes | no |
| 3. Were groups similar at baseline? | | unclear | yes | unclear |
| 4. Were participants blinded to their treatment? | | n/a | unclear | unclear |
| 5. Were treatment providers blinded? | | no | no | no |
| 6. Were groups treated the same apart from the intervention? | | yes | yes | yes |
| 7. Were outcome assessors blinded? | | unclear | unclear | unclear |
| 8. Were outcomes measured consistently across groups? | | yes | yes | yes |
| 9. Were outcomes measured reliably? | | yes | yes | yes |
| 10. Was follow-up complete, or were differences analyzed? | | n/a | n/a | n/a |
| 11. Were participants analyzed in their assigned groups? | | unclear | unclear | unclear |
| 12. Was statistical analysis appropriate? | | yes | yes | yes |
| 13. Was the trial design suitable, with deviations properly addressed? | | yes | yes | yes |
| **Grading of risk of bias**^2^ | | high | moderate | high |
| ^1^ | Quality appraisal statements from the JBI Critical Appraisal Tool for Assessment of Risk of Bias for Randomized Controlled Trials [23]. Response options for statements 1–13: yes, no, unclear, and not applicable (n/a). | | | |
| ^2^ | Risk of bias criteria as set by the authors: low (>70% “yes” answers to statements 1–13), moderate (50–70% “yes”), and high (<50% “yes”). | | | |

**Table SV.** Overview of the risk of bias assessment for the quasi-experimental studies included in the review.

| **Quality appraisal statements^1^** | | **Author (year) [reference number]** | | | | | |
| --- | --- | --- | --- | --- | --- | --- | --- |
|  |  | **Markel et al. (2018) [24]** | **Campbell et al. (1993) [26]** | **Zarei et al. (2023) [27]** | **Zarei et al. (2022) [28]** | **Wistrand et al. (2021) [29] *** | **Sandström et al. (2014) [30]** |
| 1. Is the cause-effect relationship clearly defined? | | yes | yes | yes | yes | yes | yes |
| 2. Was a control group used? | | yes | no | yes | yes | yes | yes |
| 3. Were comparison groups similar? | | yes | yes | yes | yes | yes | yes |
| 4. Did comparison groups receive similar care, aside from the intervention? | | no | no | no | no | yes | no |
| 5. Were outcomes measured multiple times before and after the intervention? | | yes | yes | yes | yes | yes | yes |
| 6. Were outcomes measured consistently across groups? | | yes | yes | yes | yes | yes | yes |
| 7. Were outcomes measured reliably? | | yes | yes | yes | yes | unclear | yes |
| 8. Was follow-up complete, or were differences analyzed? | | n/a | n/a | n/a | n/a | n/a | n/a |
| 9. Was statistical analysis appropriate? | | yes | yes | yes | yes | yes | yes |
| **Grading of risk of bias**^2^ | | low | moderate | low | low | low | low |
| ^1^ | Quality appraisal statements from the JBI Checklist for Quasi-Experimental Studies [22]. Response options for statements 1–9: yes, no, unclear, and not applicable (n/a). | | | | | | |
| ^2^ | Risk of bias criteria as set by the authors: low (>70% “yes” answers to statements 1–9), moderate (50–70% “yes”), and high (<50% “yes”). | | | | | | |
| ^*^ | To migitate potential bias, this study was independently evaluated by two external reviewers with no prior collaborations with any of the current authors. | | | | | | |
